# Dr. Bradford Augustus Richards

**Published:** 1918-12

**Authors:** 


**Dr. Bradford Augustus Richards,** McGill 1901, died at his home in Rochester, Oct. 22, of pneumonia. He specialized in eye, ear, nose and throat.

